# A paradigm for fostering patient-centered research in liver disease: The liver transplant patient-engagement program

**DOI:** 10.1097/HC9.0000000000000053

**Published:** 2023-03-17

**Authors:** Sarah R. Lieber, Lisa B. VanWagner, Alvaro Noriega Ramirez, Marina Serper, Amit G. Singal, Donna M. Evon

**Affiliations:** 1 Department of Medicine, Division of Digestive and Liver Diseases, University of Texas Southwestern (UTSW) Medical Center, Dallas, Texas, USA; 2 Division of Gastroenterology and Hepatology, Department of Medicine, University of Pennsylvania Perelman School of Medicine, Philadelphia, Pennsylvania, USA; 3 Division of Gastroenterology and Hepatology, Department of Medicine, University of North Carolina (UNC), Chapel Hill, North Carolina, USA

## Abstract

Many federal funding and regulatory agencies require patient engagement to conduct patient-centered research and drug development. We developed a liver transplantation patient-engagement program, which can serve as a model for bringing the patient perspective to digestive diseases research.

**Methods:**

Six liver transplantation patient-engagement program advisors completed training in patient engagement; participated in several virtual sessions; and completed postsession surveys.

**Results:**

Qualitative and quantitative results elucidated patient-centered liver transplantation study outcomes and barriers/facilitators to conducting clinical research. Group satisfaction was very high.

**Conclusions:**

The liver transplantation patient-engagement program model provides a paradigm for how to engage patients in the formative steps of patient-centered clinical research.

## INTRODUCTION

As metrics for high-quality care encompass more than just clinical endpoints, it is increasingly important to incorporate patient perspectives into research efforts.^1^ In the past decade, patient engagement has become a major cornerstone of research endeavors for funding agencies such as The Patient-centered Outcomes Research Institute^2^ and regulatory agencies including the Food and Drug Administration’s patient-focused drug development program.^3^,^4^ Despite the enormous value patient perspectives bring to clinical research, patient engagement has been featured in only a handful of studies on liver disease.^2^,^3^

Herein, we offer guidance on how to engage patients in grassroots, patient-driven, clinical outcomes research. We adapted previously successful patient-engagement methodologies^5^,^6^ to develop a liver transplantation patient-engagement program (LT-PEP) at the University of Texas Southwestern Medical Center. We use liver transplantation (LT) as a model, as LT survivorship goes beyond patient and graft survival to comprise the lived experiences of patients that includes symptoms, functioning, quality of life, and other patient-reported outcomes.^7^,^8^ The goal of LT-PEP was to engage the patient community, establish trustworthy bi-directional communications between transplant providers and patients, and foster research collaborations that will directly inform clinical care and improve health outcomes that matter most to LT survivors.

## PATIENTS AND METHODS

LT-PEP was founded on principles of patient engagement developed by the Patient-centered Outcomes Research Institute^2^ and modeled after a Patient Engagement Group established in 2013 at the University of North Carolina for patients with chronic hepatitis C (HCV-Patient Engagement Group).^5^,^6^ The goals of LT-PEP were to inform a transplant survivorship research agenda. Our first 5 sessions covered the following topics:

1. Identifying key concepts important to study in LT.
2. Determining the best patient-reported measures to assess these concepts.
3. Identifying barriers and facilitators to conducting clinical research with patients.
4. Reviewing research workflow, incentives, and materials (eg, flyers, consents, and interview guides).

**FIGURE 1:**
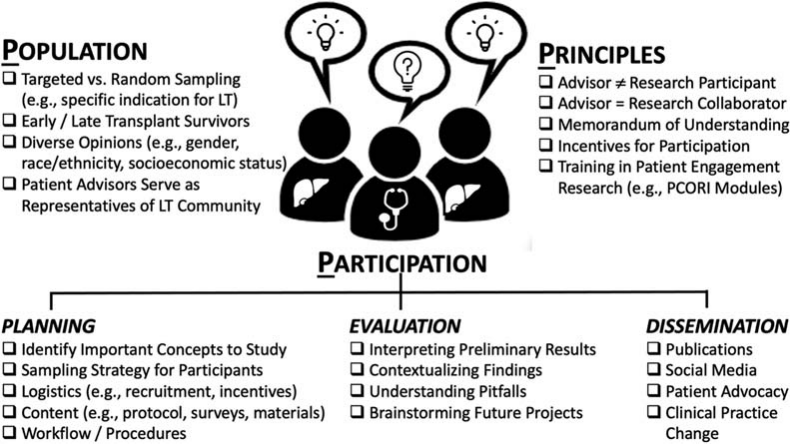
In developing a patient-engagement program, the 3P’s to consider include: (1) population: selecting the right representatives of your patient community including potentially a targeted sample of advisors; (2) principles: imparting to patient advisors the training and understanding about their role as a research collaborator; and (3) participation: various roles of patient advisors to help with research planning, interpretation of results/evaluation, and dissemination of findings.

We identified 3 essential “P’s” to devising a successful LT-PEP including consideration of the: (1) population (ie, what members of the community should be represented in an advisory panel); (2) principles that should ground patient training and discussions about roles as research advisors versus participant; and (3) participation of advisors in various activities from research planning to dissemination to the stakeholder community (Figure 1). Future sessions with advisors will contextualize preliminary data and brainstorm future research projects including interventional needs and development. IRB approval was not required to involve patients as research advisors, given they were hired as consultants with a contract. However, IRB approval was obtained to collect and publish survey and qualitative data describing LT-PEP experiences.

## RESULTS

A purposeful sampling strategy was used to select a diverse group of 6 LT survivors—33% were female patients and ages ranged from 30 to 69 years. Patient advisors included white non-Hispanic (66%), Hispanic (17%), and African American (17%) patients; indications for LT included nonalcoholic fatty liver disease (33%), hepatocellular carcinoma (33%), Hepatitis C/alcohol-associated cirrhosis (17%), and acute alcohol-associated hepatitis (17%). Advisors were compensated $50 per hour and signed a memorandum of understanding defining roles and expectations as an advisor. Advisors completed online training sessions available from Patient-centered Outcomes Research Institute (Supplemental Materials, http://links.lww.com/HC9/A135).

Five, 2-hour virtual sessions were conducted by Sarah R. Lieber and Alvaro Noriega Ramirez over a 3-month period June–September 2022 covering 4 topics outlined. LT-PEP advisors preferred virtual sessions to reduce travel requirements and infectious risk of in-person meetings. After each session, members completed an evaluation assessing quality metrics of participating in LT-PEP (Supplemental Materials, http://links.lww.com/HC9/A135).

Patients identified several concepts as the most important study outcomes to evaluate in transplant-related research including uncertainty about health, cognition, positivity, financial burden, and caregiver distress (Supplemental Figure 1, http://links.lww.com/HC9/A135). Advisors then selected survey instruments that best measured these concepts from the patient's perspective. LT-PEP advisors identified several motivations to participate in research consistent with previously published perspectives:^9^ (1) an altruist drive to help future survivors through knowledge gained from research—“desire to see a change in the transplant community” and “giving back and making the transplant experience potentially more successful for future recipients”; (2) learning more about their own progress in recovery by tracking experiences—“help with understanding the situation I am in.” Appealing to these motivations and compensating study participants for their time were recruitment and retention strategies proposed by the LT-PEP. Advisors suggested $25–$50 per 1 hour of research activity plus parking vouchers.

Advisors felt that the maximum survey burden should be ~90 minutes (ideally 30-minute sections divided into 2–3 parts). They were willing to stay an additional 60–-90 minutes after a clinic visit to participate in research. Most advisors (83%) preferred answering surveys electronically alone in a private setting, as compared with in-person. Integrating research participation seamlessly into clinical care was deemed essential. Barriers to research participation included: (1) time requirements, (2) illness/hospitalizations, (3) fatigue or other physical symptoms, and (4) obligations to work or family. To overcome barriers to research participation, LT-PEP members recommended frequent phone/text reminders, coordination around clinic/lab appointments, deadlines for data collection (eg, surveys), and reminders about the necessity of their participation to enhance knowledge about LT for the larger transplant community.

Postsession assessments demonstrated a high degree of satisfaction with participating in LT-PEP. All “strongly agreed” or “agreed” that the sessions were worth their time, their feedback will improve LT research, and they would participate in future LT-PEP sessions. Advisors were excited to participate, wanted their voices to be heard, and enjoyed connecting with other LT survivors to share stories of recovery. The use of virtual videoconferencing technology facilitated the gathering of LT-PEP advisors in a safe and efficient manner. Extra time was needed to overcome connectivity and technical issues, as well as time for storytelling and socializing, which were critical to fostering connections and trust among members. Future directions include developing patient-engagement programs for other populations including monolingual Spanish speakers, elderly populations, and caregivers.

## DISCUSSION

Standardizing processes for forming patient advisory panels will enhance patient-centered research initiatives across a broad range of gastrointestinal and liver diseases. The HCV-Patient Engagement Group and LT-PEP provide a paradigm for other liver and transplant programs to integrate patient perspectives into research endeavors (Figure 1). As highlighted here, patient-engagement programs can prioritize important areas for clinical research, identify key knowledge gaps, inform research methods, solicit patient perspectives on interventions to evaluate in future research, and help disseminate findings to the community. These patient-engagement methodologies have proven effective and can be easily adapted to other patient populations.^5^,^6^ Research teams should collaborate with patients as advisors to consult on all stages of the research cycle. Patient engagement is not simply a checkbox but requires meaningful bi-directional patient involvement.^2^ As research partners, patient stakeholders add more nuanced perspectives that directly impact patient-centeredness and clinical applicability of the research being performed.

## Supplementary Material
