# Supplementary material for: A paradigm for fostering patient-centered research in liver disease: The liver transplant patient-engagement program
Source: Hepatol Commun. 2023 Mar 17;7(4):e0053. doi: 10.1097/HC9.0000000000000053 (PMC10027036; doi:10.1097/HC9.0000000000000053)
Supplement: Supplementary file 1 [file hc9-7-e0053-s001.docx]

**SUPPLEMENTAL MATERIALS:**

1. **Trainings for Patient Advisors: Research Fundamentals Modules (**[**https://www.pcori.org/engagement/research-fundamentals**](https://www.pcori.org/engagement/research-fundamentals)**)**

- Engaging in Stakeholder-Driven Research
- [Module 1: Developing Research Questions](https://www.pcori.org/node/18437)
- [Module 2: Designing the Research Study](https://www.pcori.org/node/18438)
- [Module 3: Planning Patient-Centered Consent and Study Protocols](https://www.pcori.org/node/18439)
- [Module 4: Sampling, Recruiting, and Retaining Study Participants](https://www.pcori.org/node/18440)
- [Module 5: Understanding and Sharing Research Findings](https://www.pcori.org/node/18441)

1. **Quality Assessment of Patient Research Advisory Program:** Modeled after the Vanderbilt Community Engagement Studio Toolkit 2.0—Appendix H pages 50-52. (<https://www.meharry-vanderbilt.org/sites/vumc.org.meharry-vanderbilt/files/public_files/CESToolkit%202.0.pdf>)
